# Dynamic chromatin landscape encodes programs for perinatal transition of cardiomyocytes

**DOI:** 10.1038/s41420-023-01322-3

**Published:** 2023-01-18

**Authors:** Jing Zhang, Zhaohui Ouyang, Limei Xia, Qi Wang, Feng Zheng, Kun Xu, Yuexian Xing, Ke Wei, Shaolin Shi, Chaojun Li, Jingping Yang

**Affiliations:** 1 State Key Laboratory of Pharmaceutical Biotechnology, Medical School, Nanjing University, 210093 Nanjing, Jiangsu China; 2 Jiangsu Key Laboratory of Molecular Medicine, Medical School, Nanjing University, 210093 Nanjing, Jiangsu China; 3 Institute for Regenerative Medicine, Shanghai East Hospital, Shanghai Institute of Stem Cell Research and Clinical Translation, Shanghai Key Laboratory of Signaling and Disease Research, Frontier Science Center for Stem Cell Research, School of Life Sciences and Technology, Tongji University, 200092 Shanghai, China; 4 State Key Laboratory of Reproductive Medicine and China International Joint Research Center on Environment and Human Health, Center for Global Health, School of Public Health, Gusu School, Nanjing Medical University, 211166 Nanjing, China

**Keywords:** Chromatin remodelling, Chromatin structure, Epigenomics, Gene regulation, Reprogramming

## Abstract

The perinatal period occurring immediately before and after birth is critical for cardiomyocytes because they must change rapidly to accommodate the switch from fetal to neonatal circulation after birth. This transition is a well-orchestrated process, and any perturbation leads to unhealthy cardiomyocytes and heart disease. Despite its importance, little is known about how this transition is regulated and controlled. Here, by mapping the genome-wide chromatin accessibility, transcription-centered long-range chromatin interactions and gene expression in cardiomyocytes undergoing perinatal transition, we discovered two key transcription factors, MEF2 and AP1, that are crucial for driving the phenotypic changes within the perinatal window. Thousands of dynamic regulatory elements were found in perinatal cardiomyocytes and we show these elements mediated the transcriptional reprogramming through an elegant chromatin high-order architecture. We recompiled transcriptional program of induced stem cell-derived cardiomyocytes according to our discovered network, and they showed adult cardiomyocyte-like electrophysiological expression. Our work provides a comprehensive regulatory resource of cardiomyocytes perinatal reprogramming, and aids the gap-filling of cardiac translational research.

## Introduction

The perinatal period is a critical window for cardiomyocyte development because a significant transition of late fetal to neonatal cardiomyocytes occurs [1, 2]. This transition is characterized by a drastic decline in cell proliferation, metabolic switch, increase in extracellular matrix density and remodel of electrophysiology [3–5]. Any perturbation during this perinatal transition period can disrupt cardiomyocyte maturation. For example, premature birth has been associated with abnormal cardiomyocyte hypertrophy and cell division [6, 7]. Furthermore, engineered induced pluripotent stem cell-derived cardiomyocytes (iPSC-CMs) are proposed for disease modeling, drug testing and cell therapy. iPSC-CMs resemble mid-to-late embryonic CMs, even following extended culture [8]. As transplantation of iPSC-CMs into neonatal rat heart seems improve the maturity [9], it suggests that neonatal transition is important for maturation of iPSC-CMs. While it is clear that the perinatal transition is key to obtaining fully mature cardiomyocytes, it remains unclear how the transition is controlled. Understanding how the perinatal transition is regulated is crucial for cardiac translational research.

Similar to cardiomyocyte lineage determination and postnatal maturation, cardiomyocyte perinatal transition should be a highly coordinated process involving a well-orchestrated transcription regulation program [10, 11]. Chromatin accessibility has been found to be dynamic in both cardiomyocyte lineage determination and postnatal maturation [12–14]. Chromatin high-order architectures are known to be indispensable for cardiomyocyte development [15]. Mutation of CTCF, a crucial mediator of chromatin interactions, causes severe cardiomyocyte defects in embryos [16]. Epigenetic regulators and transcription factors that govern remodeling of these chromatin features are also known to be important for embryonic and postnatal regulation. For instance, PHF7 and GATA4 determine cardiac differentiation [17, 18] and SRF is crucial for cardiomyocyte postnatal maturation [19]. While more is known about transcription regulation in cardiomyocyte lineage determination and postnatal maturation [14, 15], less is known about the chromatin program underlying cardiomyocyte perinatal transition [20].

Here, by examining genome-wide chromatin accessibility, gene expression and chromatin high-order architectures in cardiomyocytes within the perinatal period, we identified a key transcriptional network that is critical for programming the phenotypic transition of cardiomyocytes. Our study revealed that the chromatin accessibility of the thousands of regulatory elements we identified is dynamic within the perinatal window, and the long-range interactions of these regulatory elements are remodeled in perinatal cardiomyocytes. Further analysis of the dynamic regulatory elements accommodated in the chromatin high-order architecture reveals that MEF2 and AP1 drive the downregulated and upregulated networks, respectively. Together, our study reveals a dynamic regulatory network involving transcription factors, regulatory elements and chromatin interactions that control the phenotypic transition of perinatal cardiomyocytes. We believe this new understanding of the transcriptional regulation of perinatal cardiomyocytes will aid the development of iPSC-CMs and leverage cardiac translational research.

## Results

### Chromatin accessibility reveals functional regulatory elements in perinatal cardiomyocytes

**Fig. 1:**
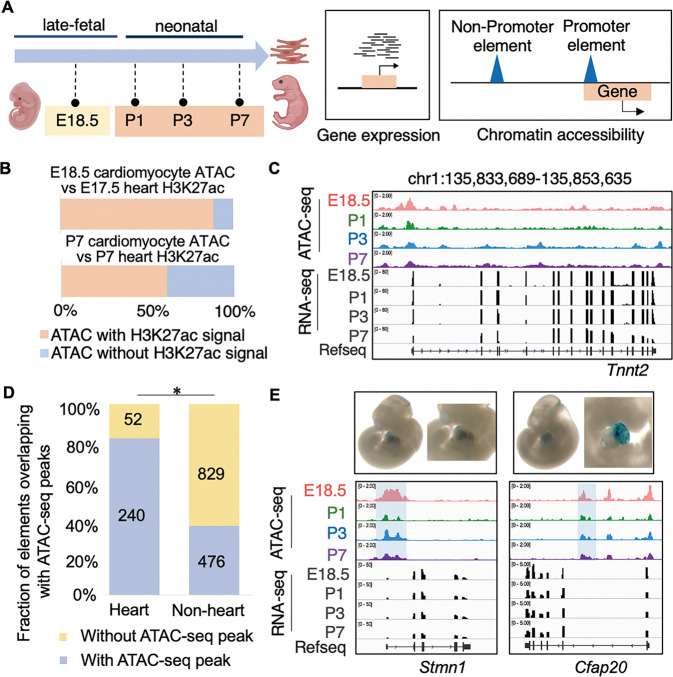
Identification of regulatory elements in perinatal cardiomyocytes. **A** Experimental strategy to identify regulatory elements in perinatal cardiomyocytes by chromatin accessibility and transcription. **B** Percentages of cardiomyocyte chromatin accessible regions with or without whole heart H3K27ac signal at late fetal or postnatal stage. **C** Genome browser view of *Tnnt2* loci with chromatin accessibility and gene expression in cardiomyocytes at four perinatal time points. **D** Fraction of heart or non-heart VISTA enhancers that overlap with cardiomyocyte chromatin accessible regions. Cardiomyocyte chromatin accessible regions are enriched in heart enhancers (240 of 292 elements) than non-heart enhancers (476 of 1305 elements) (two-tailed chi-squared test, *P* = 0, * indicates *P* < 0.05). **E** LacZ staining in mouse embryo from VISTA database reveals heart enhancer activities of the promoter region of *Stmn1* (upper left) and the non-promoter region within the *Cfap20* gene body (upper right). Genome browser view of *Stmn1* and *Cfap20* loci with chromatin accessibility and gene expression in perinatal cardiomyocytes (lower). Blue shades highlight the regions examined with LacZ staining.

To understand the regulatory program and the regulatory elements involved in the perinatal transition of cardiomyocytes, we profiled the chromatin accessibility and gene expression landscape in perinatal cardiomyocytes by ATAC-seq and RNA-seq (Fig. 1A). Cardiomyocytes were isolated from mice at four perinatal time points: embryonic day 18.5 (E18.5) of the late fetal stage, postnatal day 1 (P1), 3 (P3) and 7 (P7) of the neonatal stage. Immunofluorescence of cardiomyocyte marker cardiac troponin T (cTnT) showed high purity of the isolated cardiomyocytes even from P7 (Supplementary Fig. S1A).

The high quality and reproducible profiles (Supplementary Fig. S1B and Supplementary Table S1) revealed 63,436 accessible regions across the time points, including 16,731 at promoter and 46,705 at non-promoter regions (Supplementary Fig. S1C). When we compared these profiles with H3K27ac distribution in whole heart at each stage [21], we found that at the late embryonic stage 88.53% of the accessible regions in cardiomyocytes (E18.5) overlapped with H3K27ac regions in whole heart (E17.5) (Fig. 1B). This overlap was much lower at P7 (61%) and is likely due to lower percentage of cardiomyocytes in whole heart at P7. The chromatin accessibility profiles captured constitutively accessible regions at cardiomyocyte-specific marker gene loci such as *Tnnt2* (Fig. 1C and Supplementary Fig. S2). For genes that are not expressed in cardiomyocytes, such as endothelial cell marker *Pecam1* and fibroblast marker *Dcn* [22], their promoters were not accessible in cardiomyocytes even though the promoters displayed H3K27ac signal in P7 whole heart (Supplementary Fig. S2). These results suggested that we captured cardiomyocyte-specific chromatin features for cardiomyocyte transcription regulation.

By further examining the functionalities of these accessible regions, we found that the identified cardiomyocyte chromatin accessible regions recovered 82.2% of experimentally verified elements with heart enhancer activities in VISTA [23] (Fig. 1D). For example, the promoter region of *Stmn1* and the non-promoter region within the *Cfap20* gene body both showed high chromatin accessibility in perinatal cardiomyocytes, and these regions were consistently confirmed to be functional as assessed in transgenic mouse heart from VISTA database (Fig. 1E). In contrast, only 36.5% of the non-heart enhancers possess chromatin accessibility in cardiomyocytes (Fig. 1D). These results confirmed that our identified accessible regions are enriched for functional promoter and non-promoter regulatory elements in perinatal cardiomyocytes.

### Regulatory elements show temporal chromatin accessibility during perinatal period

**Fig. 2:**
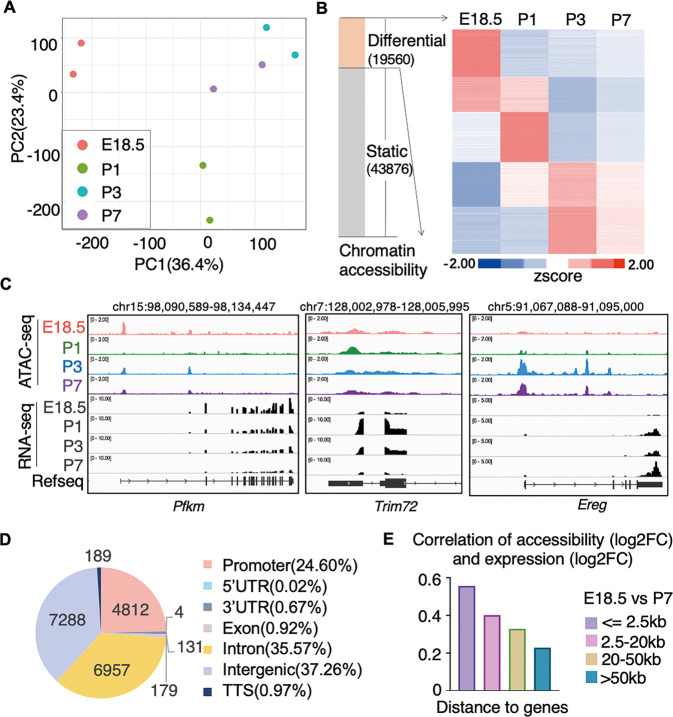
Dynamic changes of regulatory elements in perinatal cardiomyocytes. **A** Principal components analysis (PCA) of chromatin accessibility profiles. **B** Bar-chart shows the fraction of static or differential chromatin accessible regions during the perinatal window (left). Heatmap shows the dynamic chromatin accessibility of those differentially accessible regions (right). The groups of regions with decreasing or increasing accessibility after birth are marked. **C** Genome browser view of *Pfkm, Trim72* and *Ereg* loci with gene expression and chromatin accessibility in cardiomyocytes. **D** Genomic distribution of differential chromatin accessible regions in cardiomyocytes. **E** Pearson correlation between log2 fold-change (log2FC) of chromatin accessibility of these differentially accessible regions and log2FC of gene expression of their nearest differential genes in E18.5 vs. P7.

The chromatin accessibility profiles at these identified regulatory elements overall showed a distinct distribution across late fetal and neonatal stages (Fig. 2A). In total, there were 19,560 elements that showed differential accessibility at any time point by pair-wise comparisons (Fig. 2B). Clustering of these differentially accessible elements revealed groups of elements that had continuously decreasing and transiently or continuously increasing accessibility after birth (Fig. 2B). The promoter of *Pfkm* was less accessible in neonatal cardiomyocytes than in late fetal cardiomyocytes, and its transcription was accordingly downregulated. The chromatin accessibility of promoters at *Trim72* or *Ereg* showed transient or continuous increase accordingly with changes of their expression (Fig. 2C). The dynamics of chromatin accessibility at promoters was accompanied by the change of gene expression. Gene ontology and pathway analysis of genes with dynamic promoters revealed that genes with less accessible promoters were enriched for development and glucose metabolism, genes with transiently accessible promoters were involved in muscle structure development, and genes with continuously upregulated promoters were related to extracellular matrix organization (Supplementary Fig. S3A). These changes in promoter accessibility partially reflect transcriptional programming for metabolic changes and increased extracellular matrix attachment that are known to occur within this developmental window [11].

In addition to promoter regions, 75.4% of these differentially accessible regulatory elements were located at non-promoter regions (Fig. 2D). When the transcription of the nearest genes of these non-promoter regulatory elements was examined, we found that transcription was merely correlated with the accessibility of these elements. For distal elements that were 20 kb farther away from the genes, correlations were much weaker than those for differentially accessible promoters (Fig. 2E and Supplementary Fig. S3B). Taken together, these results show that chromatin accessibility of cardiomyocyte regulatory elements is temporally dynamic within the perinatal window, but the transcriptional effect of distal elements is less clear.

### Chromatin interactions mediate the transcriptional network

**Fig. 3:**
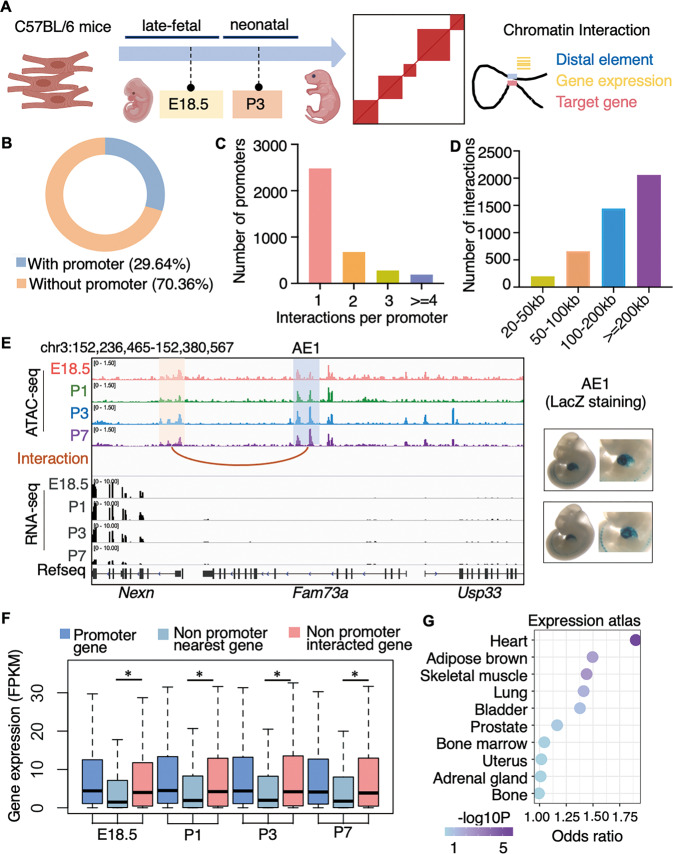
Transcriptional regulation network captured by chromatin interactions in cardiomyocytes. **A** Experimental strategy to capture chromatin interactions in perinatal cardiomyocytes. **B** Proportion of the interaction types. **C** Histogram of promoters with different numbers of interactions in HiChIP promoter-involving interactions. **D** Histogram of promoter-involving interactions with different sizes. **E** Genome browser view at *Nexn*-AE1 locus with chromatin accessibility and chromatin interaction in cardiomyocytes. The AE1 and *Nexn* promoter are marked with blue and orange boxes, respectively. The interaction between AE1 and *Nexn* promoter is indicated by the brown arch (left). LacZ staining in mouse embryo from VISTA database reveals heart enhancer activity of AE1 (right). **F** Gene expression of promoter-associated genes and the nearest or the interacting genes of the same non-promoter regulatory element (center line, median; box limits, upper and lower quartiles, Wilcoxon rank sum test, P for non-promoter nearest gene vs. interacted gene expression in E18.5, P1, P3, and P7 are 2.2E-16, 1.484E-14, 1.388E-14 and 2.511E-14, respectively. * indicates *P* < 0.05). **G** Top 10 tissues with enriched expression of target genes of the distal regulatory elements.

To comprehensively understand the regulatory roles of these dynamic elements, we used HiChIP with H3K27ac antibody to capture transcription-related chromatin 3D interactions at E18.5 and P3 (Fig. 3A and Supplementary Fig. S4A). At a resolution of 10 kb, we identified a total of 14,712 chromatin interactions in the combined HiChIP data of E18.5 and P3. Of the 14,712 interactions, 4,361 (29.64%) were with promoters (Fig. 3B) and 2,302 pairs were between promoters and distal accessible elements (Supplementary Table S2). The promoter can interact with one or more distal elements (Fig. 3C), and the sizes of these interactions ranged from 20 to 1460 kb (Fig. 3D), enabling us to investigate the regulatory role of distal elements. For instance, HiChIP detected an interaction between the promoter of *Nexn* and accessible element 1 (AE1) located 40 kb upstream (Fig. 3E). AE1 showed enhancer activity in the heart (from VISTA database). Although *Fam73a* is the nearest gene of AE1, *Nexn* that interacted with AE1 was the most highly transcribed gene in this locus (Fig. 3E). In genome-wide, the genes interacting with distal accessible elements showed significantly higher expression than genes that were nearest the elements (Fig. 3F). Furthermore, interacted target genes of the identified distal regulatory elements showed enriched expression in the heart over other tissues in the Mouse Gene Atlas (Fig. 3G). These results indicated that the chromatin architectures orchestrated a transcriptional regulatory network that is critical for perinatal cardiomyocytes.

### Integrated dynamics of chromatin accessibility and high-order organization guides the perinatal reprogramming

**Fig. 4:**
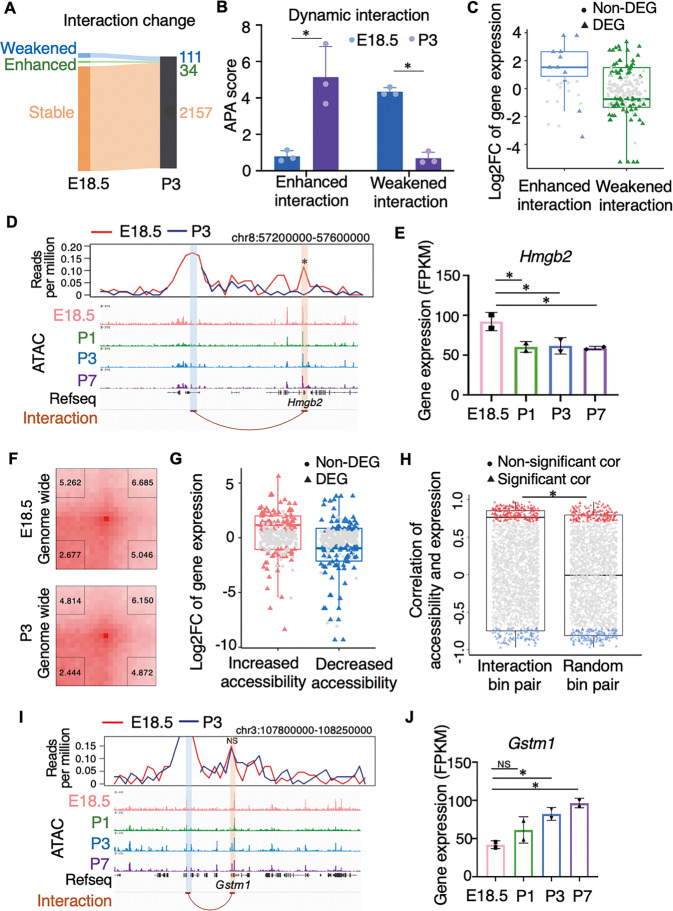
Dynamics of chromatin high-order structure mediates perinatal transcriptome. **A** Sankey diagram shows the number of chromatin interactions that are weakened, enhanced or stable from the late fetal to neonatal cardiomyocytes. **B** APA scores of dynamic interactions in the late fetal and neonatal cardiomyocytes (*n* = 3, two-tailed Student’s *t*-test, *P* = 0.0116 for APA scores of enhanced interactions in E18.5 vs. P3; *n* = 3, Wilcoxon rank sum test, *P* = 0.049 for APA scores of weakened interactions in E18.5 vs. P3. * indicates *P* < 0.05). **C** Boxplot shows dynamic interactions was accompanied by corresponding transcription changes (center line, median; box limits, upper and lower quartiles). Colored triangles represent interactions in which differential expression genes (DEG) are located. Gray dots represent interactions in which gene expression is not significantly different (Non-DEG). **D** Virtual 4C interaction profile at the distal element of *Hmgb2* in E18.5 (red line) and P3 (blue line) cardiomyocytes. Genome browser view of chromatin accessibility is aligned vertically below. The anchor (distal element) and *Hmgb2* promoter are marked with blue and orange boxes, respectively. The strength change of the interaction between them is significant (*P* = 0.002, * indicates *P* < 0.05). **E** Bar plot of gene expression (right, *n* = 2, FDR for E18.5 vs. P1, P3, and P7 are 0.033, 0.029, and 0.019, respectively. * indicates FDR < 0.05). **F** Genome-wide APA measurements of stable interactions in the late fetal and neonatal cardiomyocytes. P2LL (peak to lower left) value in the lower left corner was taken as the APA score. **G** Log2FC of differential expression genes regulated by distal regulatory elements with increased or decreased accessibility (center line, median; box limits, upper and lower quartiles). Colored triangles represent interactions in which DEG are located. Gray dots represent interactions in Non-DEG. **H** Pearson correlation of chromatin accessibility and gene expression for chromatin interactions bin pairs or randomized pairs. More pairs show positive correlation (red triangles, 204 pairs at correlation’s *P*-value < 0.05) than negative correlation (blue triangles, 103 pairs at correlation’s *P*-value < 0.05). Boxplot shows the Pearson correlation of chromatin interactions or randomized pairs with significant correlation *P*-values (center line, median; box limits, upper and lower quartiles, Wilcoxon rank sum test, *P* = 2.34E-06, * indicates *P* < 0.05, randomized pairs mean randomly matching anchors from interactions). **I** Virtual 4C interaction profile at the distal element of *Gstm1* in E18.5 (red line) and P3(blue line) cardiomyocytes. Genome browser view of chromatin accessibility is aligned vertically below. The anchor (distal element) and *Gstm1* promoter are marked with blue and orange boxes, respectively. The strength change of the interaction between them is not significant (*P* = 0.6, NS indicates not significant). **J** Bar plot of gene expression (right, *n* = 2, FDR for E18.5 vs. P1, P3, and P7 are 0.2, 0.021, and 0.005, respectively. NS indicates not significant. * indicates FDR < 0.05).

Chromatin 3D structure is highly reorganized during cardiomyocyte lineage determination [15], but it is stable in mature cardiomyocytes even under cardiac disease [24]. We then examined the chromatin structure to determine what happens during perinatal transition. Quantitative analysis of the interactions revealed that 6.3% (145 out of 2,302) of the interactions were weakened or enhanced in cardiomyocytes from the late fetal stage to the neonatal stage (Fig. 4A, B and Supplementary Table S3). These dynamic interactions coordinated with gene transcriptional regulation (Fig. 4C). For *Hmgb2*, which encodes a protein that exaggerates myocardial ischemic injury in rats [25], its promoter interacts with a distal regulatory element and this interaction was weakened after birth (Fig. 4D). Accordingly, the expression of *Hmgb2* was downregulated significantly, although the accessibility at either its promoter or the distal element was not changed (Fig. 4E). *Irs2* related distal interaction was also weakened after birth and showed downregulated gene expression (Supplementary Fig. S4B). On the other hand, the enhanced interaction between the promoter and the distal element correlated with the increased expression of *Timp3* and *Csf1* after birth (Supplementary Fig. S4C, D).

Although hundreds of chromatin interactions were dynamic, there were more chromatin interactions that were stable during the perinatal window (Fig. 4A, F). When further examined the features of these stable chromatin interactions, we observed that the chromatin accessibility at the regulatory elements in these pre-established interactions were changed at different time point. Furthermore, the changed activities of the interacting elements correlated with the transcription of their target genes (Fig. 4G). Genes interacting with distal elements were downregulated when the distal element had decreased accessibility, and vice versa. The correlations between accessibility of distal regulatory elements and transcription of their interacting genes were significantly stronger than those of random pairs (Fig. 4H). For example, the promoter of *Gstm1* stably interacted with a regulatory element located 80 kb upstream (Fig. 4I). As the accessibility of this regulatory element continuously increased in neonatal cardiomyocytes, the transcription of *Gstm1* was also upregulated accordingly (Fig. 4J). Meanwhile, the transcription of *Adamts1* was downregulated as its interacting regulatory element became inaccessible in neonatal cardiomyocytes, even though the accessibility at the *Adamts1* promoter was unchanged (Supplementary Fig. S4E). Thus, the chromatin architecture not only mediates the on/off of gene expression by targeting enhancers to promoters, but also serves as a platform to transmit delicate transcriptional instruction from distal regulatory elements.

### Key transcription factors are critical for perinatal programming

**Fig. 5:**
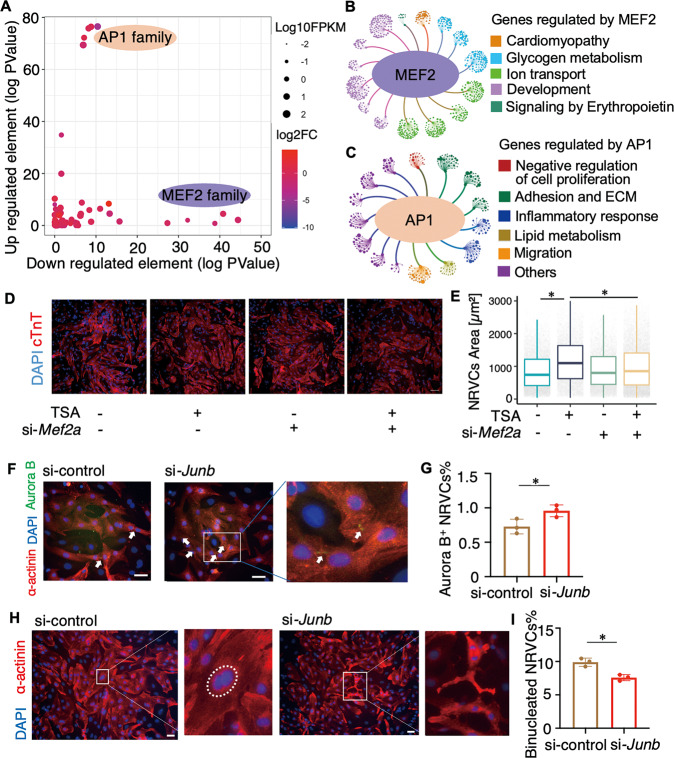
Dynamic regulatory program involves transcription factor MEF2 and AP1. **A** Motif enrichment on downregulated elements and upregulated elements in cardiomyocytes transcriptional network. **B** and **C** Network diagram of top 15 gene ontology and pathway terms associated with the genes regulated by MEF2 (**B**) or AP1 (**C**). Each node set represents a term and each node in the set represents a gene. The colors of node sets represent the term category. **D** Co-staining of cTnT and DAPI staining of NRVCs treated with TSA/si-Mef2a (–/–, +/–, –/+, +/+). Scale bars, 100 μm. The representative image is shown. **E** Quantifications of cell area (Wilcoxon rank sum test, *P* for TSA/si-*Mef2a* (–/–) vs. (+/–) and (+/–) vs. (+/+) are <2.2E-16, <2.2E-16, respectively. * indicates *P* < 0.05). **F** Co-staining of α-actinin and Aurora B of NRVCs treated with scramble siRNA or si-*Junb*. Scale bars, 100 μm. The representative image from one of three independent experiments is shown. **G** Percentage of Aurora B^+^ NRVCs (*n* = 3, two-tailed Student’s *t*-test, *P* = 0.044, * indicates *P* < 0.05). **H** Co-staining of α-actinin and DAPI staining of NRVCs treated with scramble siRNA or si-*Junb*. Scale bars, 100 μm. The representative image from one of three independent experiments is shown. **I** Percentage of binucleated NRVCs (*n* = 3, two-tailed Student’s *t*-test, *P* = 0.0063, * indicates *P* < 0.05).

As transcription factors are known to be important for chromatin function [26], we searched for potential key transcription factors that are relevant to perinatal cardiomyocyte programming. We performed motif enrichment analyses for the dynamic proximal as well as distal accessible peaks of differential expression genes. We found that motifs for the MEF2 and AP1 transcription factor families were most enriched at the elements with decreased and increased accessibility, respectively (Fig. 5A). Genes interacted with dynamic regulatory elements, which harboring MEF2 and AP1 motifs were enriched in important biological processes during the transition of perinatal cardiomyocytes (Fig. 5B, C). For example, genes regulated by MEF2 are involved in biological pathways as glucose metabolism (Fig. 5B and Supplementary Fig. S5A) and phenotype ontology as CMs electrophysiology (Supplementary Fig. S5C). Meanwhile, genes regulated by AP1 are involved in cell proliferation and extracellular matrix (Fig. 5C and Supplementary Fig. S5B, C).

To examine the role of the established regulatory program in cardiomyocyte perinatal transition, we manipulated the activity of the identified transcription factors in isolated neonatal rat ventricular cardiomyocytes (NRVCs). As the expression level of MEF2 is not decrease significantly but MEF2 activity was inhibited significantly by HDAC after birth [27], we used trichostatin A (TSA), an inhibitor of HDAC, to control the activity of MEF2. Treatment of TSA, which could reactivate the downregulated MEF2 elements, changed the morphology of the neonatal cardiomyocytes as the cell area increased significantly (Fig. 5D, E), indicating cardiomyocyte hypertrophy [28]. When the transcriptome of NRVCs with and without TSA treatment were compared, it was confirmed that the expression of MEF2 family members did not change significantly (Supplementary Fig. S6A), but MEF2-regulated-genes were reactivated after TSA treatment (Supplementary Fig. S6B). This is consistent with what happened in heart disease [29, 30]. We found that the MEF2-regulated hypertrophy genes were also reactivated under transverse aortic constriction-induced hypertrophy in mouse cardiac model (GSE188762) [31] (Supplementary Fig. S6C and Table S4). As TSA could not specifically activate MEF2 activity, we further tested the dependency on MEF2A of the TSA induced hypertrophy. We found the treatment of si-*Mef2a* could significantly reduce the abnormal cell area (Fig. 5D, E). These results demonstrate that MEF2 and MEF2-regulated-genes are relevant for perinatal transition and might be reactivated in mature cardiomyocytes under disease conditions.

To understand the role of upregulated AP1-motif-harboring-elements, we used siRNA to reduce the expression of *Junb*, the most highly expressed member of the AP1 complex, in NRVCs (Supplementary Fig. S6D, E). We examined the cellular phenotypes related to the function of AP1-regulated-genes, such as cell proliferation (Fig. 5C). 5-Ethynyl-2’-deoxyuridine (EdU) staining showed that DNA synthesis was unchanged in treated cells (Supplementary Fig. S6F, G). However, the fraction of Aurora B-positive and phospho-Histone H3 (pHH3)-positive NRVCs increased significantly (Fig. 5F, G and Supplementary Fig. S6H, I), indicating increased cytokinesis. Furthermore, the number of binuclear NRVCs decreased significantly (Fig. 5H, I). We also examined the role of another AP1 complex member *Atf3*, and the results confirmed the effect of *Atf3* on cytokinesis as *Junb* (Supplementary Fig. S6J–L). These results indicate that the upregulated AP1-elements in NRVCs could stop cardiomyocyte cytokinesis in postnatal cardiomyocytes, the most significant change during the perinatal transition.

### iPSC-CMs with remodeled MEF2-program show adult cardiomyocyte-like electrophysiology

**Fig. 6:**
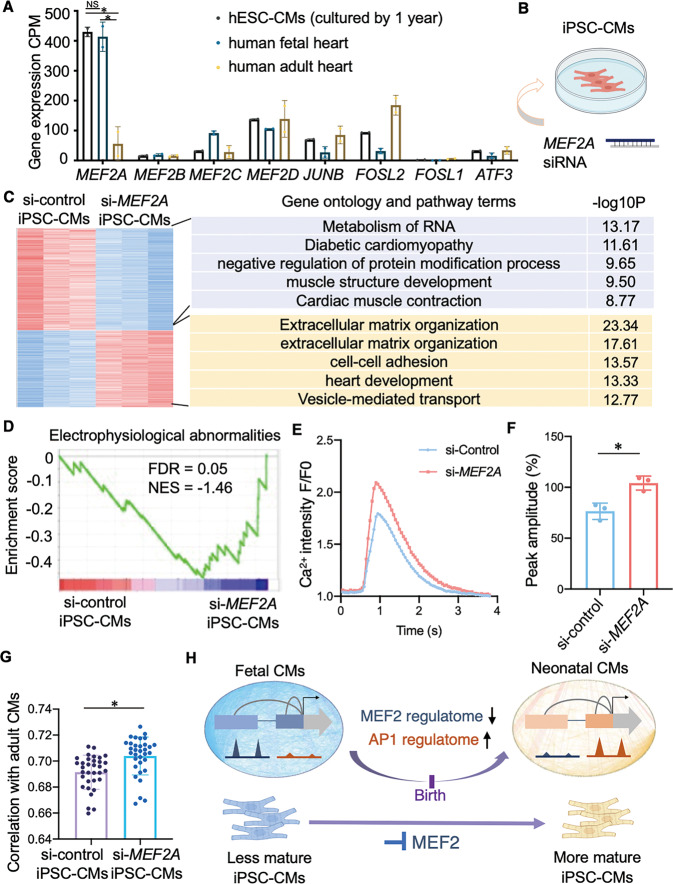
Inhibition of *MEF2A* improves the similarity of iPSC-CMs to in-vivo adult cardiomyocytes. **A** MEF2 and AP1 family members gene expression (CPM) in hESC-CMs, human fetal and adult heart from GSE62913 (*n* = 2, FDR for hESC-CMs vs. fetal, hESC-CMs vs. adult, and fetal vs. adult of *MEF2A* expression are 1, 1.051E-17, 2.369E-13, accessed using edgeR. * indicates FDR < 0.05. NS indicates not significant). **B** Schematic diagram of *MEF2A* siRNA processing iPSC-CMs. **C** Heatmap shows the differentially genes expression (*P* < 0.01) of iPSC-CMs treated with scramble siRNA or si-*MEF2A* (left). Enriched gene ontology and pathway terms for genes located at each cluster in heatmap along with their –log10(*P*-values) (right). **D** GSEA enrichment analysis shows that the expression of genes associated with electrophysiological abnormalities is downregulated after the inhibition of *MEF2A* in iPSC-CMs. **E**, **F** Representative Ca^2+^ transients (E) and quantification of relative Ca^2+^ transient amplitude (**F**) in iPSC-CMs treated with scramble siRNA or si-*MEF2A* (*n* = 3, two-tailed Student’s *t*-test, *P* = 0.005, * indicates *P* < 0.05). **G** Spearman correlation between iPSC-CMs and adult CMs transcriptome (iPSC-CMs treated with scramble siRNA, *n* = 3; iPSC-CMs treated with si-*MEF2A*, *n* = 3; adult CMs, *n* = 11; Wilcoxon rank sum test, *P* = 8.029E-05, * indicates *P* < 0.05). **H** Graph chart summarizes the regulatory reprogramming in perinatal cardiomyocytes. Key transcription factors, MEF2 and AP1, mediate the remodeling of regulatory program in perinatal CMs. The manipulation of *MEF2A* would induce the iPSC-CMs more like adult CMs.

The in-vitro iPSC-CMs exhibit less mature characteristics [11, 20, 32] and these less mature phenotypes would affect their application as drug testing [33, 34]. We tried to utilize the perinatal regulation mechanism to promote the maturation of iPSC-CMs. We first examined the expression of the key transcription factors revealed by our study in transcriptome of induced CMs [35]. The results showed that *MEF2A* is abnormally highly expressed in human in-vitro induced CMs as that in human fetal but not in adult (Fig. 6A). We then used *MEF2A*-siRNA to reprogram the regulatome in iPSC-CMs (Fig. 6B and Supplementary Fig. S7A). We found that inhibition of *MEF2A* in iPSC-CMs resulted in significant downregulation of gene expression associated with cardiomyopathy, which was consistent with the terms associated with MEF2-regulated-genes discovered in perinatal transitions (Fig. 6C and Supplementary Fig. S5A).

Among the cardiomyopathy, we further found that the inhibition of *MEF2A* in iPSC-CMs resulted in significant change of gene expression associated with electrophysiological abnormalities (Fig. 6D). Electrophysiological features are major measurements for cardiac translational research of iPSC-CMs [4]. The critical pacemaker gene *HCN4*, which is important for synchronized beating [11], is highly expressed in engineered cultured CMs and fetal CMs, but was downregulated in adult CMs (Supplementary Fig. S7B). Interestingly, we found that inhibition of *MEF2A* could significantly decrease the transcription of *HCN4* in iPSC-CMs (Supplementary Fig. S7C). Additionally, Ca^2+^ handling is one of the major hallmarks of cardiomyocytes maturation [11]. We found that decreased MEF2A expression in iPSC-CMs significantly increased the amplitude of Ca^2+^ transient (Fig. 6E, F). Furthermore, iPSC-CMs with inhibition of *MEF2A* showed increased overall correlation with adult CMs samples (Fig. 6G). All these results revealed the manipulation of *MEF2A* would induce the iPSC-CMs more like in-vivo adult CMs, which will contribute to cardiovascular research fields.

Together, our results suggest that cardiomyocyte perinatal transition is tightly controlled by transcription regulation, which involves transcription factors and 1D to 3D chromatin architectures. Key transcription factors such as MEF2 and AP1 are crucial for the remodeling of regulatory elements within the chromatin architecture to govern the programming of the perinatal transition, and recompiling of MEF2 regulation plays a role in the improvement of iPSC-CMs maturity (Fig. 6H).

## Discussion

Understanding how cardiomyocytes are programmed to develop and mature is important for cell engineering, disease modeling, drug testing and cell therapy. However, how the transcription regulation program operates within the critical perinatal window during development is poorly understood. Here, we explored this transcriptional regulation by integrating a multilayer chromatin landscape.

Our results showed that the chromatin interaction provides a basic framework for the regulatory elements to transmit their regulatory signal for programming during the perinatal window. The dynamics of chromatin interactions mediate changes in transcriptional regulation and phenotypic variations. Proper chromatin architecture is necessary for perinatal cardiomyocyte homeostasis. For example, *Hmgb2* is downregulated postnatally through a decrease in the interaction between its promoter and the distal regulatory element after birth to introduce a proper perinatal transition of cardiomyocytes. The abnormally increased expression of *Hmgb2* after birth exaggerates myocardial ischemic injury in rats [25]. Although many of the identified interactions are not changed during the perinatal transition, they mediate the transcriptional regulation of important genes for cardiomyocyte health. Any damage to this framework would greatly affect transcription programming. It has been reported that cardiomyocyte-specific depletion of CTCF, an essential factor for chromatin interactions, leads to spontaneous and sustained cardiac dilation as well as the heart failure stress response [24]. Thus, proper chromatin architecture is subjected to precise regulation in cardiomyocytes during the perinatal period.

We discovered key transcription factors driving the dynamic activities of regulatory elements in chromatin architecture. We identified a MEF2-mediated regulatory program that is downregulated in the perinatal window. MEF2 is considered a central cardiac transcription factor [27] and has been reported to be essential for viability and mitochondrial function in cardiomyocytes [36, 37]. While MEF2 is essential for the identity of cardiomyocyte both before and after birth, its regulatory activity is downregulated at certain regulatory elements postnatally to obtain healthy cardiomyocytes. Consistent with our observations, abnormal activation of MEF2 by CaM kinase signaling in adults leads to cardiomyopathy as cardiomyocytes undergo hypertrophy [29, 30]. In contrast, repression of MEF2 and its regulated network in iPSC-CMs lead to a change of electrophysiology. Thus, although MEF2 is required for cardiomyocyte identity, ectopic activation of MEF2 can perturb their proper regulatory roles during perinatal transition and damage the health and function of cardiomyocytes.

Notably, that we discovered an upregulated program driven by the transcription factor AP1. This AP1-mediated regulatory program controls multiple facets of perinatal phenotypic changes, such as cell cycle exit and lipid metabolism. Consistent with our finding that AP1 drives the upregulated program, a recent study also showed that fetal-to-adult gained accessible regions in human cardiomyocytes are enriched for AP1 motifs [14], but the role of AP1 in human cardiomyocytes was not further investigated. Our results reveal that the upregulated AP1 in neonatal cardiomyocytes inhibits cardiomyocyte cytokinesis and increases binucleation, which are well-known phenotypic changes in perinatal cardiomyocytes. Although this appears to be contradictory to the report that AP1 stimulates cardiomyocyte proliferation in zebrafish heart regeneration [38], it indicates that the regulatory role of AP1 is different in vertebrates with or without cardiomyocyte regenerative capacities. It was recently reported that AP1-associated regeneration-responsive enhancers, as *inhba* enhancer in regeneration-competent teleost fish are evolutionarily changed in mammals and fail to promote regeneration [22]. Thus, AP1-associated regulation is evolutionarily preserved in both regeneration-competent and regeneration-incompetent animals, but the role is redirected from usage for regeneration in fish cardiomyocytes to cytokinesis inhibition in mammalian perinatal cardiomyocytes.

In summary, we captured the chromatin remodeling in cardiomyocytes during the perinatal transition. Our results show that dynamic regulatory programs are important for the programming of cardiomyocyte phenotypic transition. The data resource and findings in our study would provide knowledge to leverage the development of better approaches to promote iPSC-CMs maturity. We believe that careful manipulation of the cardiomyocyte epigenome to mimic perinatal regulation could potentially offer a route to therapies for heart disease.

## Materials and methods

### Isolation of cardiomyocytes

Animal experiments were carried out with wild-type C57BL/6 mice (Model Animal Research Center of Nanjing University) or Sprague Dawley rats (Shanghai Laboratory Animal Center, CAS). Mice were males and females with ages at embryonic day 18.5, postnatal days 1, 3, and 7. Rats were males and females with ages at postnatal days 1. Animals were killed using CO_2_ asphyxia and the cardiomyocytes from mice or rats were isolated rapidly from left ventricles as described [39]. The tissues were cut into pieces, and then digested in 0.25% trypsin (Gibco, 15090046) at 4 °C for 12–16 h. The tissues were then transferred to a new tube and incubated with 0.1% collagenase Type II (Worthington, LS004176) and 1% BSA (MP biomedicals, 9048–46–8) in the reciprocating shaker bath at 115 rpm, 37 °C. The cell suspensions were collected every ~8 min for 3 times, and then passed through a 100-μm cell strainer (Millipore, SLHP035RB). Cell suspensions were centrifuged for 5 min at 300 × *g*. The cells were resuspended with DMEM HG medium (Gibco, 12800-017) with 10% FBS (Gibco, 10270–1106), seeded onto uncoated 100-mm plastic dishes and incubated for 1.5–2 h in 5% CO_2_ humidified atmosphere incubator. The cardiomyocytes in supernatant were collected and the non-cardiomyocytes, which attached to dishes were discarded. Cardiomyocytes were used for following experiments directly or seeded onto plates for treatment.

### Preparation of iPSC-CMs

Human induced pluripotent stem cell (hiPSC) line ZSSY001 (RC01001-A) was kindly provided by Nuwacell [40]. hiPSCs were plated on 6-well plates coated with Matrigel (Corning, 354277) and maintained with Essential 8 ^TM^ Medium (Gibco, A15169-01, A15171-01). At ~80% confluence, Essential 8^TM^ Medium was replaced with inducing medium: RPMI 1640 (Gibco, 11875-093)/B-27 minus insulin Medium (Gibco, A18956-01) with 6 µM CHIR-99021 (Sellect, S1263), to begin the process of differentiation (Day 0). After 2 days culture in the inducing medium, medium was replaced with RPMI 1640/B-27 minus insulin Medium (Day 2). On Day 3, medium was changed to RPMI/B-27 minus insulin Medium with 5 μM IWP2 (Sellect, S7065). After 48 h of treatment with IWP2, medium was changed to RPMI 1640/B-27 minus insulin Medium (Day 5). At Day 7, medium was changed to RPMI 1640/B-27 Medium (Gibco, A17504-044), followed by medium change every 48 h with RPMI 1640/B-27 Medium. Beating iPSC-CMs could become apparent between day 7 and 11 of differentiation. At ~Day 13, RPMI 1640/B-27 Medium was substituted with CSM Medium (ScienCell, 5911, 5962) for 72 h to metabolically purify iPSC-CMs. Purified iPSC-CMs were then digested into single-cell suspension by TrypLE (Gibco, A12177-01) and then were plated on 12-well or 96-well plates coated with matrigel.

### ATAC-seq library construction

ATAC-seq was performed as described previously [41]. In brief, 50,000 live cells were washed in cold PBS buffer and lysed in buffer with 0.01% Digitonin (Promega, G9441), 0.1% NP40 (Sigma, 11332473001) and 0.1% Tween-20 (Sigma, 11332465001). Transposition was performed at 37 °C, 1000 rpm for 30 min with Tn5 transposase (Vyzame, TD501). After purification of the DNA with Zymo DNA Clean and Concentrator 5 columns (Zymo Research, D4013), the material was pre-amplified for 5 cycles. Additional PCR cycles were evaluated by real-time PCR and final product was cleaned by AMPure XP Beads (Beckman, A63880) at a 1× ratio. HiSeqX10 sequencer (Illumina) was used to sequence the library with 150 bp paired-end reads mode at Annoroad Gene Technology Company (Beijing, China). Two biological replications were carried out for each time point.

### Analysis of ATAC-seq

#### Peak calling and annotation

For each sample, reads were trimmed for adaptors with cutadapt [42] version 1.18 and aligned with bowtie2 [43] version 2.3.0 to mm10 with parameters “-no-mixed”, “-no-discordant”. SAMtools [44] version 1.3.1, MarkDuplicates from Picard, and bedtools version 2.25.0 were used to remove duplications in aligned reads. MACS2 [45] version 2.1.2 was used for peak calling and with parameters “-SPMR” to normalize the bedgraph files to million reads. Then we removed peaks that overlapped blacklist from ENCODE consortium [46]. Genomic features were annotated using “annotatePeak” in HOMER [47] version 4.7 with default parameters. Promoter regions were defined as 2.5 kb around RefSeq TSS of protein-coding genes. H3K27ac peaks in whole heart [21] were obtained from the Gene Expression Omnibus (GEO) under the accession GSE52386. Overlapping between accessible regions in cardiomyocyte and H3K27ac peaks in whole heart was performed with GenomicRanges [48] version 1.36.1.

#### Identification of differential peaks

DiffBind [49] version 2.12.0 was used to obtain the peak occupancy (TMM normalized score) and perform differential analysis on all samples. Spearman correlation coefficient was calculated for chromatin accessibility between biological replicates for each time point using “cor” in R. The principal component analysis (PCA) plot was generated using TMM normalized score. Differential accessible regions were identified as FDR less than 0.01 and abs(log2FC) greater than 1 for each pair-wise comparison. Differential accessible peaks across all comparisons were combined. Z-scores were calculated based on TMM normalized scores for each region and then K-means cluster analysis was performed using Cluster 3.0 and visualized with TreeView [50]. Pearson correlation was calculated for log2FC of chromatin accessibility and log2FC of gene expression using “cor” in R.

### HiChIP library construction

The HiChIP was performed as previously described [51] with the following modifications. Approximately 3 million cells were used for each experiment. Cells were fixed with 1% formaldehyde (Sigma, F8775-25ML). Nuclei were isolated with 1% SDS, and digested using 400U MboI (NEB, R0147) at 37 °C overnight. After ligation, we sheared the chromatin on Sonics VCX-130 with 15 s on and 30 s off for 3 cycles. 4 μg H3K27ac antibody (Abcam, ab4729) was used. The chromatin–antibody complex was captured with Protein G beads (Invitrogen, 10004D). Libraries were constructed for HiChIP samples with NEBNext Ultra DNA Library Prep Kit for Illumina (NEB, E7370). HiSeqX10 sequencer (Illumina) was used to sequence the library with 150 bp paired-end reads mode. Three biological replications were carried out for each time point.

### Analysis of HiChIP

#### HiChIP data processing

For each sample, reads were trimmed for adaptor with cutadapt version 1.18 and aligned to mm10 genome using the HiC-Pro pipeline [52] version 2.8.0. Default settings were used to remove duplicate reads, assign reads to MboI restriction fragments, filter for valid interactions, and generate binned interaction matrices.

#### Identification of chromatin interactions

Interactions were called using Fit-Hi-C [53] version 2.0.7 based on matrix_file for each sample or for combined matrix by running the following command: “hicpro2fithic.py –i matrix_file -b abs.bed -s iced.matrix.biases -o prepared-fithic-data -r 10000” and “fithic -i interactionCounts.gz -f fragmentMappability.gz -t biases.gz -o fithic-output -L 10000 -U 300000000 -r 10000”. The combined matrix file was obtained by combining hic_files of all samples using HiC-Pro. Interactions were filtered to keep with interactions with *P*-value less than 1e-4, distances between 20 kb and 2 Mb and interactions count >8. Virtual 4C data were extracted with the Juicebox tool “dump”. Pearson correlation of replicates was calculated based on the ice normalized read counts and log10(normalized counts) was plotted using the “smoothScatter” function in R.

#### Identification of differential chromatin interactions

The counts for interaction in each sample were extracted from the matrix file in HiC-Pro output. The differential interactions between time points were called using edgeR [54] version 3.26.8 with the default normalization method. Differential interactions were identified as *P*-value less than 0.05 and abs(log2FC) greater than 1.

#### Aggregate peak analysis

Aggregate peak analysis (APA) [55] was performed using Juicer tools version 0.8 by running the following command: “juicer_tools_linux_0.8.jar apa -r 10000 hic_file interaction_file output_file -u”. P2LL (Peak to Lower Left) value was taken as the APA score. APA score >1 suggests consistent focal enrichment of interaction contacts that fall within the center of the matrix [24, 55].

### RNA-seq library preparation and sequencing

mRNA was purified from total RNA using poly-T oligo-attached magnetic beads. Sequencing libraries were generated using NEBNext UltraTM RNA Library Prep Kit for Illumina (#E7530L, NEB, USA) following the manufacturer’s instruction. HiSeqX10 sequencer (Illumina) was used to sequence the library with 150 bp paired-end reads mode.

### RNA-seq analysis

The adaptors-trimmed and quality-filtered reads were mapped to mm10, rn6 using HISAT2 [56] version 2.1.0 with default parameters. Transcript assembly was performed using StringTie version 1.3.5 [57]. “genomecov” scale function from “bedtools” to normalized RNA-seq as read count per million for visualization. Expression levels were also measured as fragments per kilobase of exon model per million mapped fragments (FPKM). Differential expression was analyzed using edgeR version 3.26.8. Adjust *P*-values (FDR) were calculated by using “p.adjust” in R. Differential expressed genes were identified as FDR less than 0.05 and abs(log2FC) greater than 1. RNA-seq for hESC-CMs, human fetal and adult heart is downloaded from the Gene Expression Omnibus (GEO) under the accession GSE62913 [35] and differential expression also was analyzed using edgeR version 3.26.8. For transcriptome correlation analysis, human adult CMs transcriptome was obtained from the Gene Expression Omnibus (GEO) under the accession GSE156702 [14]. Spearman correlation coefficient between iPSC-CMs and adult CMs transcriptome was calculated using “cor” in R.

### TSA and si-*Mef2a* treatment in NRVCs

One day after plating, NRVCs were treated without or with the TSA (ApexBio, A8183) or DMSO (MP biomedicals, 196055), and transfected with 50 nM siRNA negative control (Sangon Biotech) or siRNA against *Mef2a* (Sangon Biotech). After 12 h of transfection, Opti-MEM (Gibco, 31985-070) was replaced with new medium. Cells were fixed or collected after 48 h.

### siRNAs transfection

One day after plating, cells were transfected with 50 nM scramble siRNA (Sangon Biotech), si-*MEF2A* (Sangon Biotech), si-*Junb* (Sangon Biotech) or si-*Atf3* (Sangon Biotech) using Lipofectamine® 2000 Reagent (Invitrogen, 11668). The sequence of the scramble siRNA is 5’-UUCUCCGAACGUGUCACGU-3’. The sequence of si-*MEF2A* is 5’-CUCUAACAAACUGUUUCAAUA-3’. The sequence of si-*Junb* is 5’-CCCUGGCAGUCUUUCUCUU-3’. The sequence of si-*Atf3* is 5’-ACACUCUCCAGUUUCUCUG-3’. After 12 h of transfection, Opti-MEM (Gibco, 31985-070) was replaced with respective culture medium of the cells.

### Ca^2+^ handling

The culture medium was removed and the cells were washed three times with PBS. Then 2 μM Fluo-4 AM working solution (Beyotime, S1060) was added. Cells were incubate at 37°C for 30 min for fluorescence probe loading. After washing with PBS for 3 times and incubation for 20–30 min, the fluorescence of Fluo-4 was detected by laser confocal microscope to determine the change of intracellular calcium ion concentration.

### Quantitative real-time polymerase chain reaction (qRT-PCR)

Total mRNA was extracted using TRIzol RNA Isolation Reagents (Thermo, 15596018) following manufacturer’s instruction. The RNA obtained (200 ng) was reverse-transcribed using PrimeScript™ RT reagent Kit (Takara, RR047A) following the manufacturer’s instruction. mRNA levels for *MEF2A* and *Junb* were measured by qRT-PCR using TB Green Premix Ex Taq™ (Takara, RR420A) and CFX384 Real-Time Systems (Bio-Rad) according to the manufacturer’s instruction. The primers of *Gapdh* (*Rattus*) are: 5’-GACATGCCGCCTGGAGAAAC-3’ (forward), 5’-AGCCCAGGATGCCCTTTAG-3’ (reverse). The primers of *Junb* (*Rattus*) are: 5’-TACAAACTCCTGAAACCCACCT-3’ (forward), 5’-TCCCTGACCCGAAAAGTAGC-3’ (reverse). The primers of *Atf3* (*Rattus*) are: 5’-GATTCGCCATCCAGAACAAGC-3’ (forward), 5’-GCAATTTTGTTTCTTTCCCGC-3’ (reverse). The primers of *GAPDH* (*Homo*) are: 5’-GTGGACCTGACCTGCCGTCT-3’ (forward), 5’-GGAGGAGTGGGTGTCGCTGT-3’ (reverse). The primers of *MEF2A* (*Homo*) are: 5’-ATGAAAGCAGAACCAACTCGG-3’ (forward), 5’-CCAGGTGCGATTTTATGATTCC-3’ (reverse).

### Transcription factor motif enrichment analysis

Motif enrichment analysis were based on dynamic promoters and dynamic distal ATAC-seq elements involved in the chromatin interactions. We used HOMER version 4.7 to perform motif analysis with mm10 genome as background. The results were visualized with the ggplot2 version 3.1.1. Motif scan for a specific transcription factor was performed using Homer by running the following command: “scanMotifGenomeWide.pl TF.motif (download from Homer softwore) mm10 -bed > TF.motif.bed”. Then dynamic regulatory elements harboring the MEF2 or AP1 motifs were used to construct the TF regulatome.

### Gene enrichment analysis

Target genes in the regulatory network were defined as genes associated with accessible promoters or genes interacted with distal accessible regions. Expression enrichment on tissues in the Mouse Gene Atlas was performed with Enrichr (https://maayanlab.cloud/Enrichr). Human Phenotype Ontology enrichment was performed with g:Profiler (https://biit.cs.ut.ee/gprofiler/gost). MEF2 or AP1-regulated-genes were defined as differential genes associated with proximal or distal differential elements with MEF2 or AP1 motif. Gene ontology and pathway analysis were performed with Metascape [58] version 3.5 (http://metascape.org). The results were visualized with the ggplot2 version 3.1.1 and Gephi version 0.9.2. The enrichment of gene set as all genes regulated by MEF2 on transcriptome of NRVCs, hypertrophy genes regulated by MEF2 on transcriptome of transverse aortic constriction-induced hypertrophy model or electrophysiology abnormity genes regulated by MEF2 on transcriptome of iPSC-CMs were performed using GSEA [59] version 3.0.

### Immunofluorescence staining

Cells were fixed with 4% paraformaldehyde (BBI Life Science, EB06FA0001) for 1 h, permeabilized with 0.5% Triton X-100 (Dingguo, AR-0341) for 1 h, and blocked in 2% BSA (Yeasen, 36101ES76) for 2 h. Cells were then stained for 2 h with the following primary antibodies diluted in 2% BSA solution in PBS: anti-α-actinin (1:500; Sigma, A7811), anti-pHH3 (1:500; CST, #9706), anti-cTnT (1:500; abcam, ab64623 for rat cardiomyocytes; Thermo, MS-295-P0 for mouse cardiomyocytes), anti-Aurora B (1:500; abcam, ab2254). Cells were washed with PBS and incubated for 1 h with the secondary antibodies: Donkey anti-Rabbit IgG (H + L), DyLight 594 (1:200; Thermo, SA5-10040), Donkey anti-Goat IgG (H + L), DyLight 594 (1:200; Thermo, SA5-10088), Donkey anti-mouse IgG (H + L), DyLight 650 (1:200; Thermo, SA5-10169) and stained with DAPI (Mpbio, 157574). EdU stainings were performed using the Click-iT® EdU Alexa Fluor® 488 Imaging Kit (Thermo, C10337) according to the manufacturer’s instructions. Images were acquired by fluorescence microscopy (Zeiss, Axio Vert A1) or High-content fluorescence microscopy (PerkinElmer, Opera Phenix).

### Quantification of immunofluorescence

Cell areas, length-to-width ratio, and the percentages of EdU^+^ NRVCs were obtained using Harmony 4.8. The numbers of Aurora B^+^ NRVCs, pHH3^+^ NRVCs and binucleated NRVCs were obtained by counting the number of cells with positive signals, and the percentages were calculated by dividing the number of signal positive cells by the total number of cells in each image.

### Statistical analysis

The following statistical tests were performed or otherwise described in bioinformatics analysis: two-tailed Student’s *t*-test on the data passing normality assessment and equal variance for Figs. 4B, 5G, I, 6F, Supplementary Figs. S6G, I–L, S7A, non-parametric Wilcoxon rank sum test was used to analyze data in abnormal distribution for Figs. 3F, 4B, H, 5E, 6G, Supplementary Fig. S6E and two-tailed chi-square test for Fig. 1D. All experiments were conducted on at least two biological replications. Data on Figs. 4B, E, J, 5G, I, 6F, G, Supplementary Figs. S4B–E, S6A, D, E, G, I–L, S7A–C are mean ± SD.

## Supplementary information

Supplemental Figures

Supplementary Table S1

Supplementary Table S2

Supplementary Table S3

Supplementary Table S4

## Data Availability

All data needed to evaluate the conclusions in the paper are present in the paper and/or the Supplementary Materials. RNA-seq, ATAC-seq and HiChIP-seq data of perinatal CMs and NRVCs have been deposited at GEO (GSE178673) with security token ‘upizqqyijvgvbox’. RNA-seq data of iPSC-CMs have been deposited at GEO (GSE196655) with security token ‘mdqjmcwglrafdoz’.
