# Supplementary material for: Dynamic chromatin landscape encodes programs for perinatal transition of cardiomyocytes
Source: Cell Death Discov. 2023 Jan 18;9:11. doi: 10.1038/s41420-023-01322-3 (PMC9849264; doi:10.1038/s41420-023-01322-3)
Supplement: Supplementary file 1 — Supplemental Figures [file 41420_2023_1322_MOESM1_ESM.pdf]

Supplemental Materials

Dynamic chromatin landscape encodes programs for perinatal transition of cardiomyocytes

Jing Zhang, Zhaohui Ouyang, Limei Xia, Qi Wang, Feng Zheng, Kun Xu, Yuexian Xing, Ke Wei, Shaolin Shi, Chaojun Li, Jingping Yang

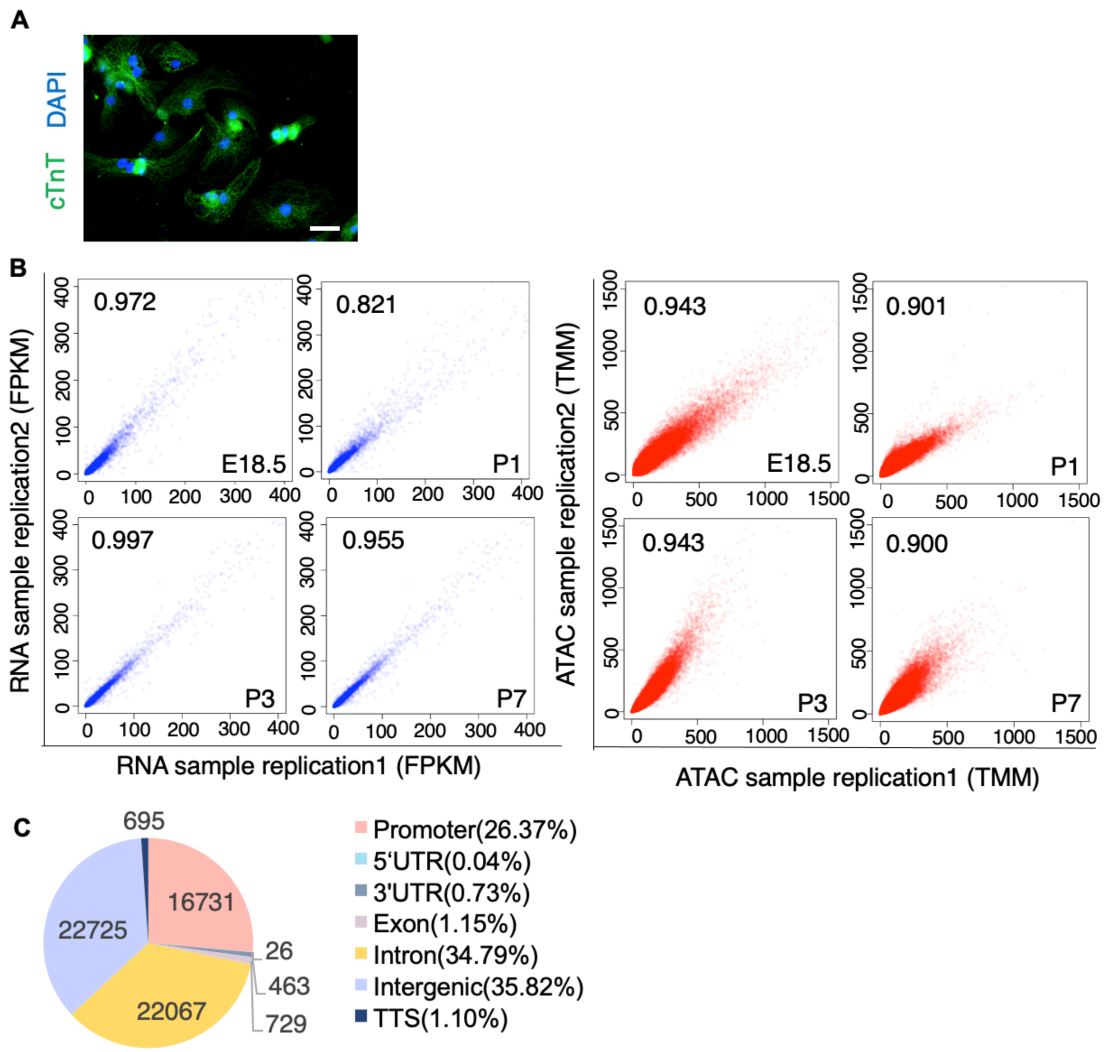

Figure S1. Quality control of RNA-seq and ATAC-seq. A cTnT staining of cardiomyocytes isolated from P7. Scale bars, 100 µm. The representative image from one of the three independent experiments is shown. **B** Scatterplot of the gene expression from RNA-seq between biological replicates for each time point (left). Scatterplot of the chromatin accessibility between biological replicates for each time point (right). Spearman correlation coefficient is marked in the upper left corner. **C** Genomic distribution of all chromatin accessible regions across the perinatal time points in cardiomyocytes.

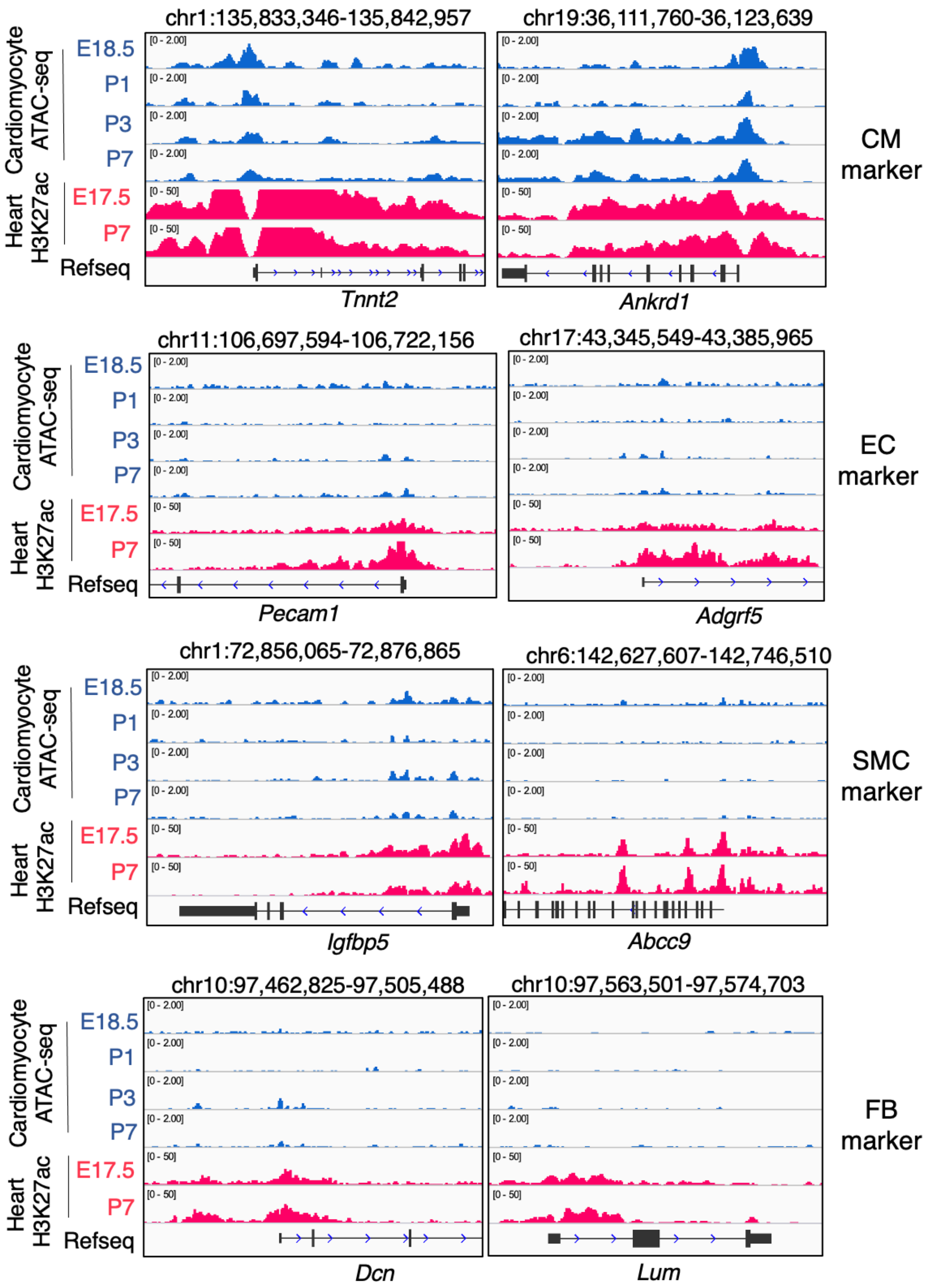

Figure S2. Visualization of cardiomyocyte or non-cardiomyocyte marker gene loci. Genome browser view of cardiomyocyte or non-cardiomyocyte marker gene loci with tracks for chromatin accessibility in cardiomyocytes at four perinatal time points and H3K27ac signal in whole heart at two perinatal time points. CM, cardiomyocyte; EC, endothelial cell; SMC, smooth muscle cell; FB, fibroblast.

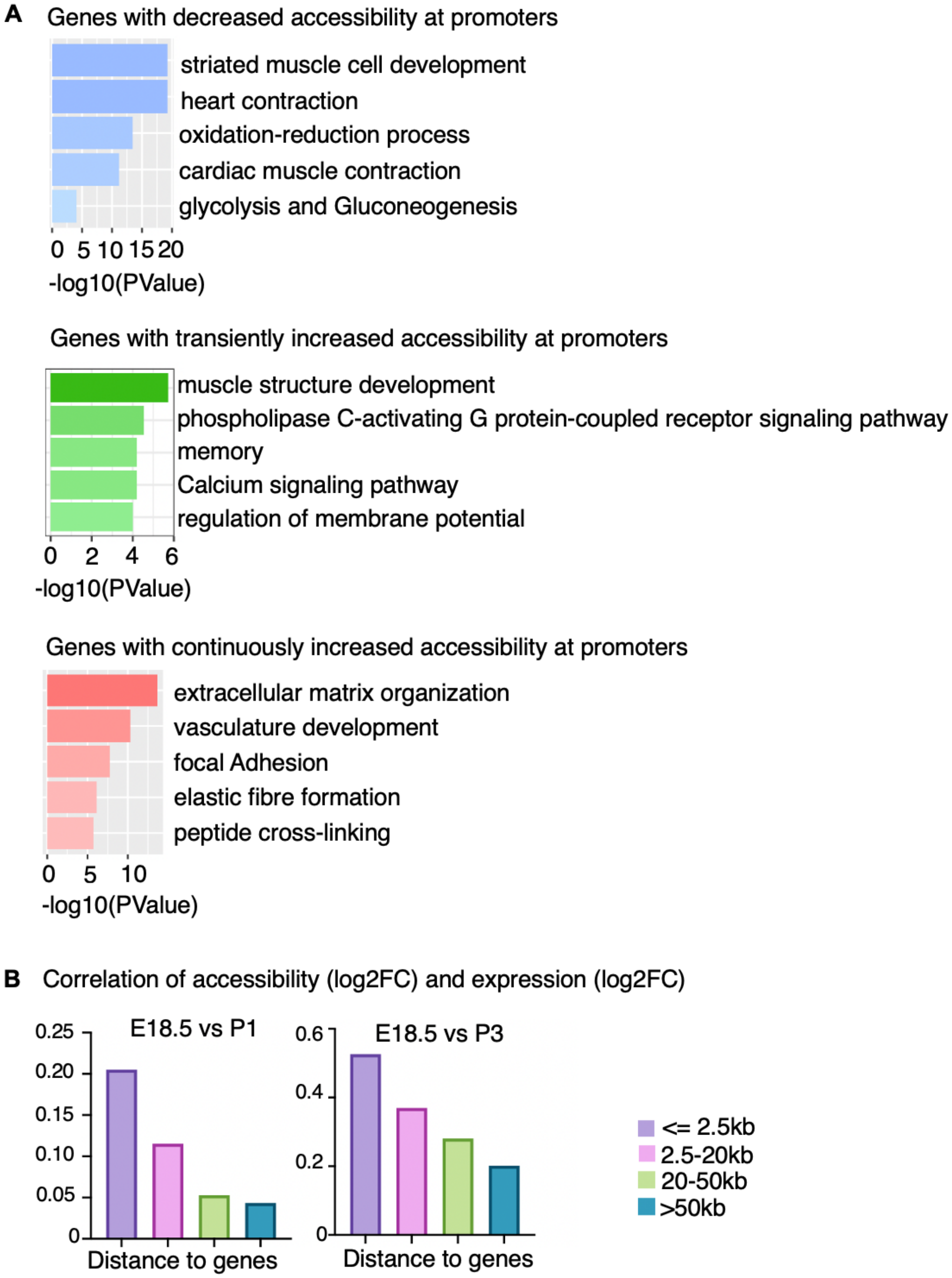

Figure S3. Differential chromatin accessibility of perinatal cardiomyocytes. A Top 5 enriched gene ontology and pathway terms on genes with down-regulated promoters (top) or genes with transiently up-regulated promoters (middle) and continuously up-regulated promoters (bottom). **B** Pearson correlation between log2 fold change (log2FC) of chromatin accessibility of these differentially accessible

regions and log2FC of gene expression of their nearest differential genes in E18.5 vs P1 (left) and E18.5 vs P3 (right).

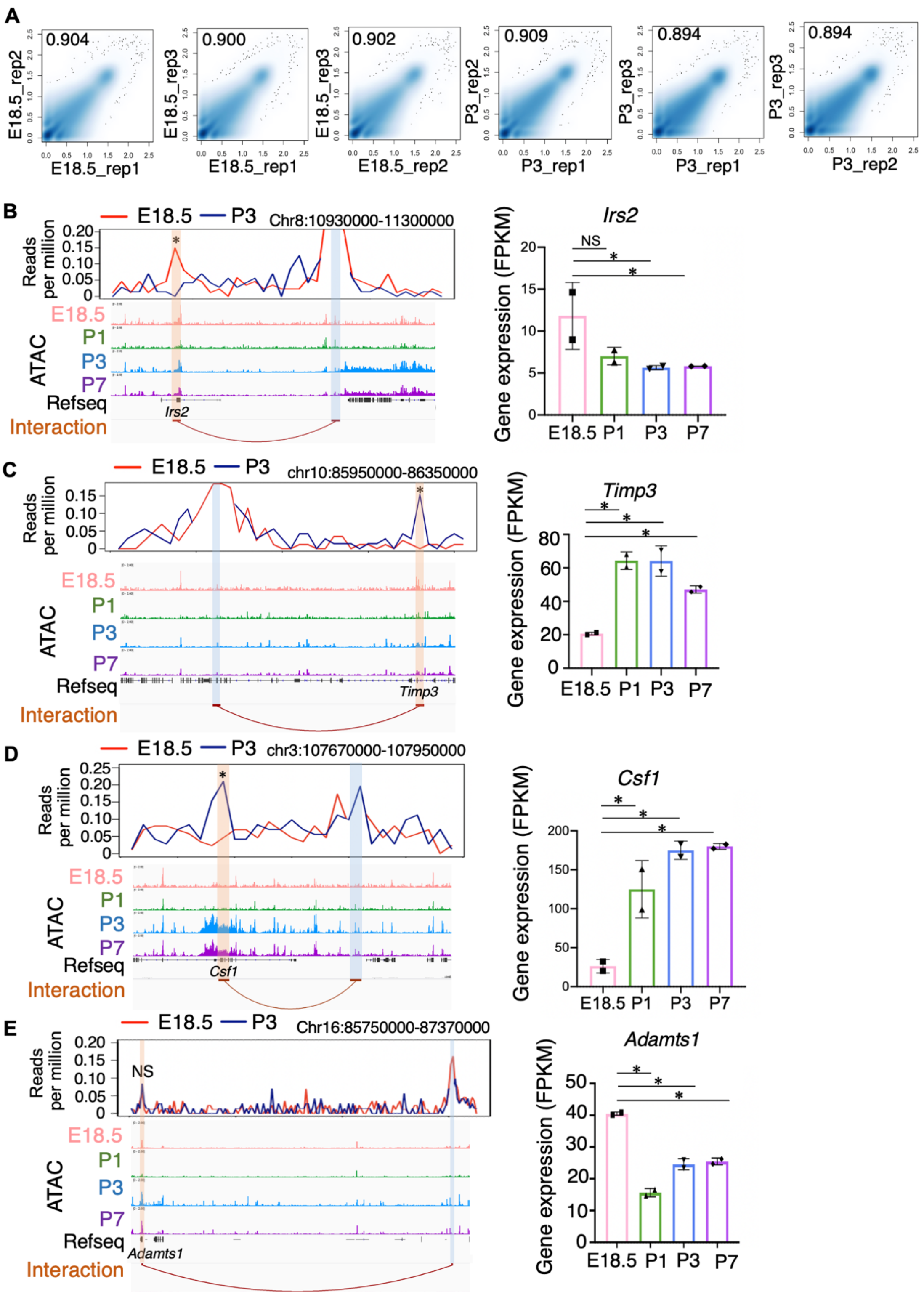

Figure S4. Chromatin architecture mediates transcriptional regulation of cardiomyocytes. **A** Scatterplot of the interaction contacts from Hi-ChIP between biological replicates for each time point. Pearson correlation coefficient is marked in the upper left corner. **B-E** Virtual 4C interaction profile at the distal element of *Irs2*

(B), *Timp3*(C), *Csf1* (D), *Adamts1* (E) in E18.5 (red line) and P3 (blue line) cardiomyocytes. Genome browser view of chromatin accessibility is aligned vertically below. The anchor (distal element) and gene promoter are marked with blue and orange boxes, respectively. The strength change of the interaction between them is significant at *Irs2*, *Timp3*, *Csf1* locus (left, P= 0.0003, 0.008, 0.021, respectively. * indicates P<0.05) and unchanged at *Adamts1* locus (left, P= 0.8, NS indicates not significant). Bar plot of gene expression (right, n=2, FDR of *Irs2* gene expression for E18.5 vs P1, P3, and P7 are 0.051, 0.01, and 0.01, those of *Timp3* are 0.0004, 0.0004, and 0.0009, *Csf1* are 0.0008, 0.0003, and 0.0001, *Adamts1* are 0.0003, 0.002, and 0.003. * indicates FDR<0.05, NS indicates not significant).

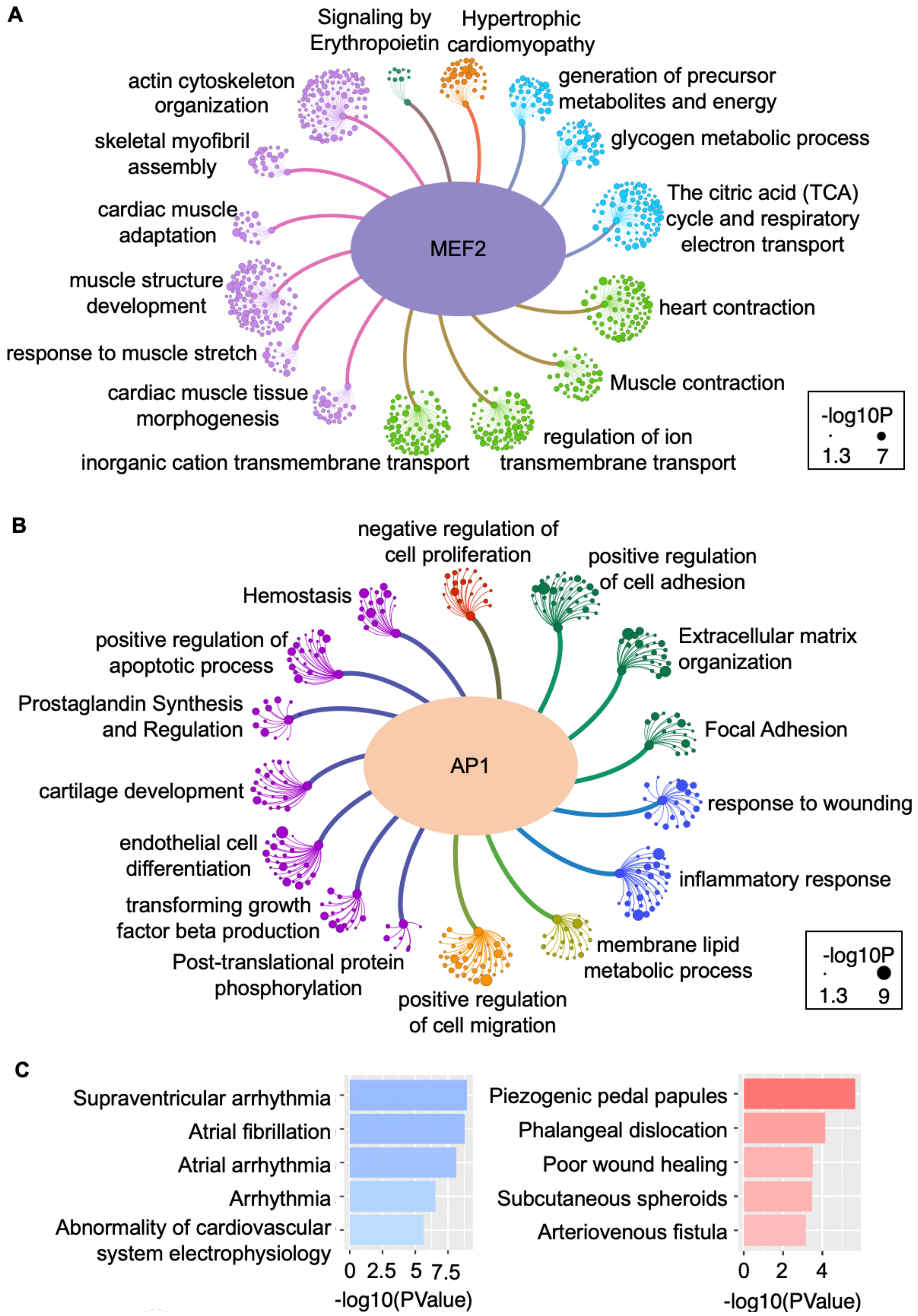

Figure S5. Perinatal cardiomyocyte transition is controlled by MEF2 and AP1. A and B Network diagram of top 15 enriched gene ontology and pathway terms on genes regulated by MEF2 (A) and AP1 (B). Each node set represents a term and each node in the set represents a gene. The colors of node sets represent the term category.

The node sizes are the -log10P of differential gene expression. **C** Top 5 Human Phenotype Ontology terms enriched for target genes regulated by MEF2 (left) and AP1 (right).

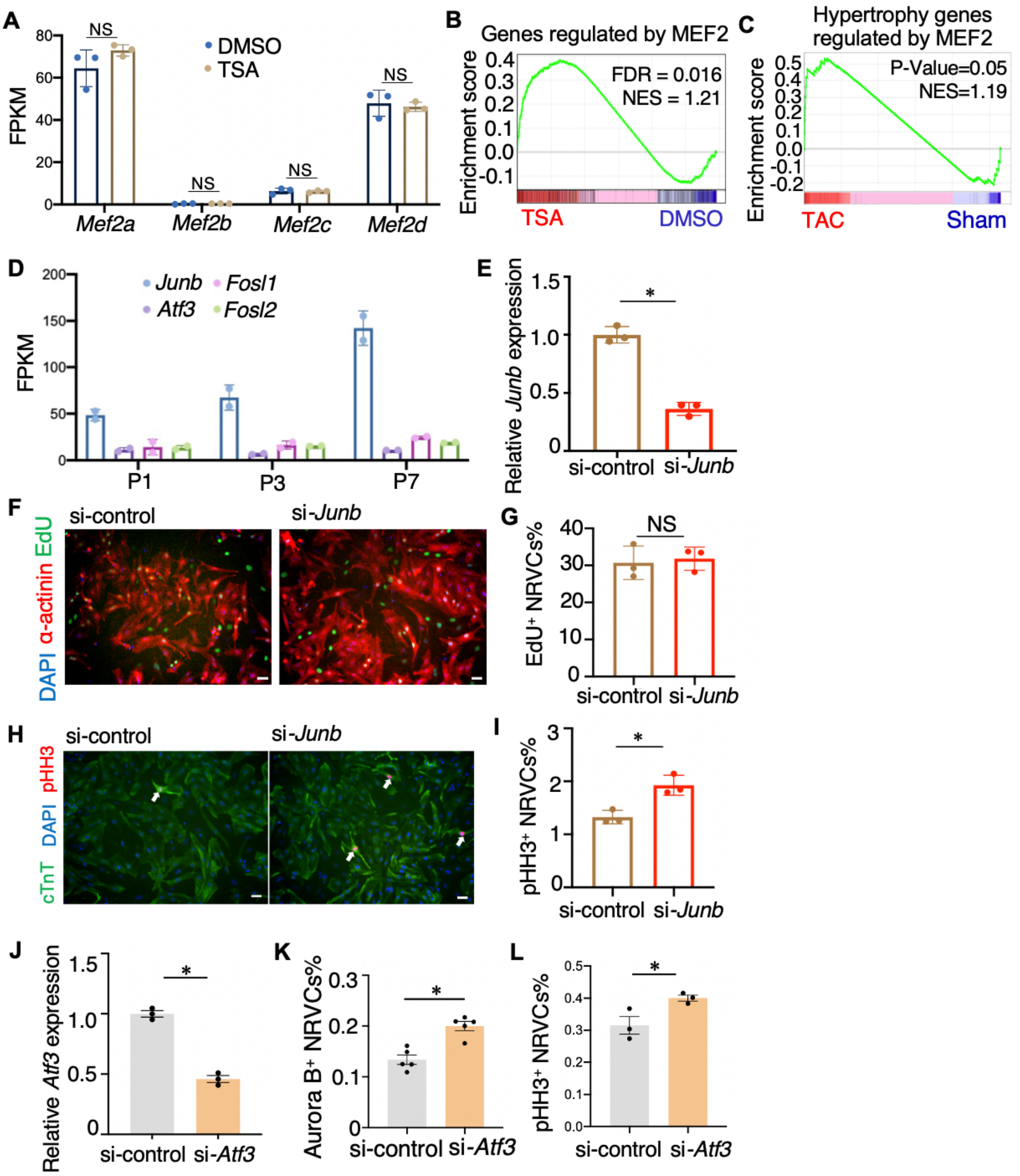

Figure S6. MEF2 and AP1 drive the phenotypic changes within the perinatal window. **A** MEF2 family members gene expression (FPKM) in NRVCs treated with DMSO or TSA (n=3, FDR for *Mef2a*, *Mef2b*, *Mef2c,* and *Mef2d* expression in DMSO vs TSA are 0.808, 1, 1, and 0.974 respectively. NS indicates not significant). **B** GSEA analysis of genes regulated by MEF2 in transcriptome of NRVCs treated with DMSO or TSA. TSA treatment reactivates genes regulated by MEF2. **C** GSEA analysis for MEF2-regulated hypertrophy genes under the transverse aortic constriction-induced hypertrophy in mouse cardiac model. **D** Gene expression of AP1 complex members in

postnatal CMs. **E** Relative *Junb* expression in NRVCs treated with scramble siRNA or siRNA against *Junb* (n=3, Wilcoxon rank sum test, P=0.049, * indicates P<0.05). **F** Co-staining of α-actinin and EdU of NRVCs treated with scramble siRNA or si-*Junb*. Scale bars, 100 μm. The representative image from one of three independent experiments is shown. **G** Percentage of EdU$^+$ NRVCs (n=3, two-tailed Student's t test, P= 0.743, NS indicates not significant). **H** Co-staining of cTnT and pHH3 of NRVCs treated with scramble siRNA and si-*Junb*. Scale bars, 100 μm. The representative image from one of the three independent experiments is shown. **I** Percentage of pHH3$^+$ NRVCs treated with scramble siRNA and si-*Junb*. (n=3, two-tailed Student's t test, P=0.01, * indicates P<0.05). **J** Relative *Atf3* expression in NRVCs treated with scramble siRNA or siRNA against *Atf3* (n=3, two-tailed Student's t test, P= 0.0001, * indicates P<0.05). **K** Percentage of Aurora B$^+$ NRVCs treated with scramble siRNA or si-*Atf3* (n=5, two-tailed Student's t test, P=0.025, * indicates P<0.05). **L** Percentage of pHH3$^+$ NRVCs treated with scramble siRNA and si-*Atf3*. (n=3, two-tailed Student's t test, P=0.021, * indicates P<0.05).

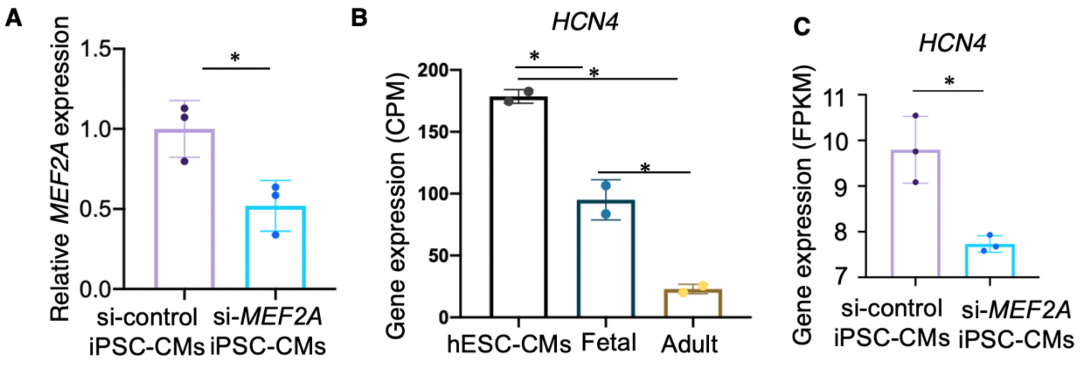

Figure S7. Inhibition of *MEF2A* expression in iPSC-CMs. A Relative *MEF2A* expression in iPSC-CMs treated with scramble siRNA or siRNA against *MEF2A* (n=3, two-tailed Student's t test, P=0.0249, * indicates P<0.05). **B** Gene expression of *HCN4* in hESC-CMs, fetal and adult CMs. (n=2, FDR for hESC-CMs vs fetal, hESC-CMs vs adult, and fetal vs adult are 0.002, 1.562E-13, 0.0004, respectively. * indicates FDR<0.05). **C** *HCN4* expression in iPSC-CMs treated with scramble siRNA or si-*MEF2A* (n=3, FDR for iPSC-CMs treated with scramble siRNA vs si-*MEF2A* is 0.343, P=0.0051, * indicates P<0.05).
